# Synaptic plasticity and roles of orexin in distinct domains of the olfactory tubercle

**DOI:** 10.3389/fncir.2024.1473403

**Published:** 2024-11-07

**Authors:** Sajib Podder, Yoshihiro Murata, Mutsuo Taniguchi, Shogo Shimizu, Masahiro Yamaguchi

**Affiliations:** Department of Physiology, Kochi Medical School, Kochi University, Kochi, Japan

**Keywords:** synaptic plasticity, glutamatergic transmission, orexigenic neuromodulator, olfactory cortex, olfactory memory

## Abstract

Olfactory behavior is highly plastic, and the olfactory tubercle (OT), a component of the olfactory cortex and ventral striatum, includes anteromedial (amOT) and lateral (lOT) domains with roles in attractive and aversive olfactory behavioral learning, respectively. However, the underlying properties of synaptic plasticity in these domains are incompletely understood. Synaptic plasticity is regulated by multiple signals including synaptic inputs and neuromodulators. Interestingly, the amOT domain exhibits high expression of various receptors for neuromodulators. We investigated synaptic plasticity in mouse OT slices by combining electrical stimulation and treatment with the appetite-promoting neuropeptide orexin, the receptors of which are highly expressed in the amOT. In both the amOT and lOT, one round of 2-Hz burst stimulation elicited short-term potentiation of the field excitatory postsynaptic potential, whereas three rounds of stimulation induced long-term potentiation (LTP) that persisted for 150 min. In the amOT, orexin-A induced LTP was blocked by the orexin receptor type 1 antagonist SB334867. Orexin-A also facilitated LTP induction in the amOT by one round of 2-Hz burst stimulation. By contrast, these effects were not observed in the lOT. These results highlighted the similarity and difference in synaptic plasticity between the OT domains and suggested that orexin facilitates synaptic plasticity in the amOT during olfactory learning processes such as food odor learning.

## 1 Introduction

Olfactory behavior is highly plastic, and it is generally linked to emotion and motivation. Previous studies identified critical brain regions for plastic changes in odor-guided motivated behaviors. In the olfactory cortex, the olfactory tubercle (OT), also termed the tubular striatum (Heimer, 1978; Wesson and Wilson, 2011; Wesson, 2020), is involved in plastic behavioral changes. The OT participates in the olfactory experience-dependent acquisition of behaviors (Gadziola et al., 2015, 2020; Zhang et al., 2017; Millman and Murthy, 2020).

The OT integrates peripheral inputs from the olfactory bulb and central inputs from the intracortical areas and sends major outputs to the ventral pallidum, a subregion of the ventral basal forebrain complex that regulates emotions, motivation, and motivated behaviors (Heimer, 1978; Wesson and Wilson, 2011; Yamaguchi, 2024). Several lines of evidence have shown that the OT serves as a crucial hub for odor-guided motivated behaviors (FitzGerald et al., 2014; Dibenedictis et al., 2015; Gadziola et al., 2015). We previously demonstrated that distinct domains of the OT become activated following attractive or aversive odor learning (Murata et al., 2015). The anteromedial OT domain (amOT) is activated by odor conditioned with a food reward, and the lateral OT domain (lOT) is activated by odor conditioned with a foot-shock punishment. Subsequent studies also showed the involvement of the medial OT in odor-attractive behaviors (Dibenedictis et al., 2015; Zhang et al., 2017). Our recent study using optogenetics identified structural changes of synaptic boutons in the amOT and lOT by associating the activation of synaptic inputs with reward or punishment (Sha et al., 2023). These results suggested that OT domain-specific modifications of synaptic efficacy, i.e., synaptic plasticity, underlie the mechanisms of odor-guided motivated behaviors.

Synaptic plasticity is regulated by multiple signals including synaptic inputs and neuromodulators. Orexin, also known as hypocretin (de Lecea et al., 1998), is an appetite-stimulating neuropeptide that facilitates motivated and reward-seeking behaviors (Boutrel et al., 2013; Mahler et al., 2014; Milbank and López, 2019). Orexin neurons are mainly localized in the hypothalamus, and they project to various brain regions including olfactory cortical regions (Peyron et al., 1998). Orexin has two receptor subtypes, namely orexin receptor type 1 (OxR1) and 2 (OxR2), which are abundantly expressed in the brain (Sakurai et al., 1998). The OT expresses orexin receptors in rodents (Hervieu et al., 2001; Cluderay et al., 2002; Caillol et al., 2003). Our previous study using qRT-PCR indicated that the mRNA expression of *Hcrtr1* (OxR1) and *Hcrtr2* (OxR2) were higher in the amOT than in the lOT, and the extent was greater in *Hcrtr1* than in *Hcrtr2* (Nogi et al., 2020). This characteristic expression of *Hcrtr1*, together with the general notion that reward-related behaviors are more closely associated with OxR1 activation than with OxR2 activation (Mahler et al., 2014), suggests the functional significance of orexin for odor-guided attractive behaviors through OxR1 signaling in the amOT.

These observations suggest that the OT has substantial synaptic plasticity, and that orexin is involved in the synaptic plasticity of the amOT that underlies odor-guided attractive behaviors. Thus, we examined whether long-term potentiation (LTP) of excitatory synaptic transmission, a cellular mechanism of learning and memory, occurs in the OT, by measuring excitatory postsynaptic potential (EPSP) in mouse OT brain slices and investigating the effect of orexin on synaptic plasticity.

## 2 Materials and methods

### 2.1 Animals

All experimental procedures were conducted according to the Physiology Society of Japan guidelines and were approved by the Kochi Medical School Animal Care and Use Committee. International guidelines were followed to reduce the number of animals used. C57BL/6N mice (Japan SLC Inc., Shizuoka, Japan) were bred and housed in the Kochi Medical School animal facility. Before experimentation, these mice were housed in plastic cages with wood shavings as bedding and granted *ad libitum* access to food and water.

### 2.2 Acute brain slice preparation

Male mice at age of 25-35 days were used for preparing acute brain slices according to our previous LTP studies in the olfactory bulb of young mice (Namba et al., 2016) and rats (Tong et al., 2017). We previously reported that OT domain-specific activation was already evident in mice at postnatal day 21 (Murofushi et al., 2018). A male mouse was anesthetized with isoflurane and sacrificed to dissect the brain. A hemisphere of the brain without the olfactory bulbs, cerebellum and brainstem was embedded in 5% agarose in HEPES buffer (pH 7.4) to prepare coronal sections including the OT (300 μm thick) of Paxinos coordinates from 5.78 mm interaural and 1.98 mm bregma to 4.30 mm interaural and 0.50 mm bregma in sucrose-modified artificial cerebrospinal fluid (ACSF) containing 200 mM sucrose, 25 mM NaHCO_3_, 3 mM KCl, 1 mM NaH_2_PO_4_, 3.6 mM MgCl_2_, 15 mM glucose, and 0.5 mM CaCl_2_ (pH 7.4, saturated with 95% O_2_ and 5% CO_2_) using a vibratome (700smz, Campden Instruments, England). The OT slices were incubated in normal ACSF containing 125 mM NaCl, 25 mM NaHCO_3_, 3 mM KCl, 1 mM NaH_2_PO_4_, 1 mM MgCl_2_, 15 mM glucose, and 2 mM CaCl_2_ (pH 7.4, saturated with 95% O_2_ and 5% CO_2_) for 1 h at 32°C. Subsequently, one of the OT slices was transferred to a recording chamber for electrophysiological experiments. The recording chamber was perfused continuously using normal ACSF at a flow rate of 1 mL/min throughout the experiment.

### 2.3 Electrophysiology

**FIGURE 1:**
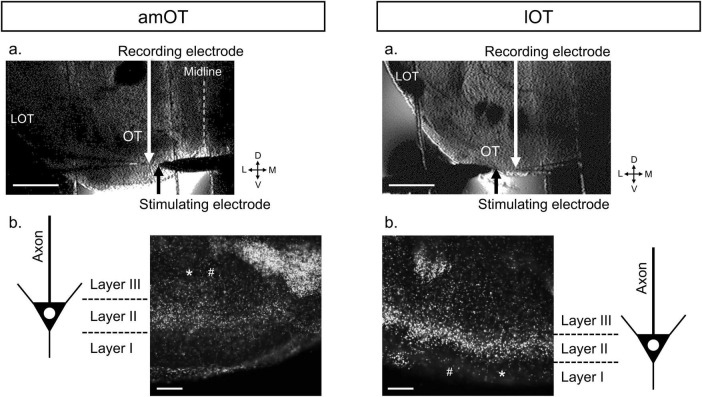
Representative coronal brain slice preparations (300 μm thick) including the amOT or lOT during and after fEPSP recordings. **(a)** Infra-red differential interference contrast images of brain slices during fEPSP recordings. LOT, lateral olfactory tract; D, dorsal; L, lateral; V, ventral; M, medial. Scale bar, 500 μm. **(b)** DAPI staining of the brain slices after the recordings. The DAPI images were merged with the bright-field images of tissue destruction by the recording (*) and stimulating (#) electrodes to identify the electrode positions in the layer structure of the OT. In this case, we identified the recording and stimulating electrodes in layer III of the amOT (left) and layer I of the lOT (right). Scale bar, 100 μm.

**FIGURE 2:**
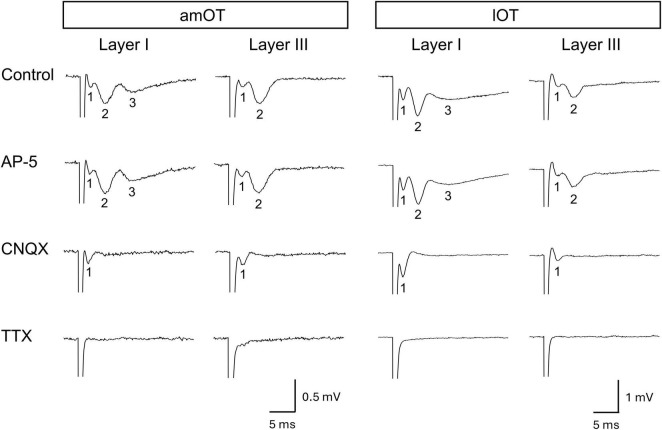
Evoked field potentials in the OT. Representative recording traces and their pharmacological characteristics are presented in columns. Each column presents a recording set from the same brain slice. Each brain slice was obtained from an individual mouse. The recording traces in the control present the weak negativity (field-potential component 1) followed by one (layer III) or two (layer I) slow deflections (field-potential components 2 and 3). Bath application of drug: NMDA receptor antagonist AP-5 (50 μM), AMPA and kainate receptor antagonist CNQX (30 μM), or voltage-gated sodium channel blocker TTX (1 μM).

**FIGURE 3:**
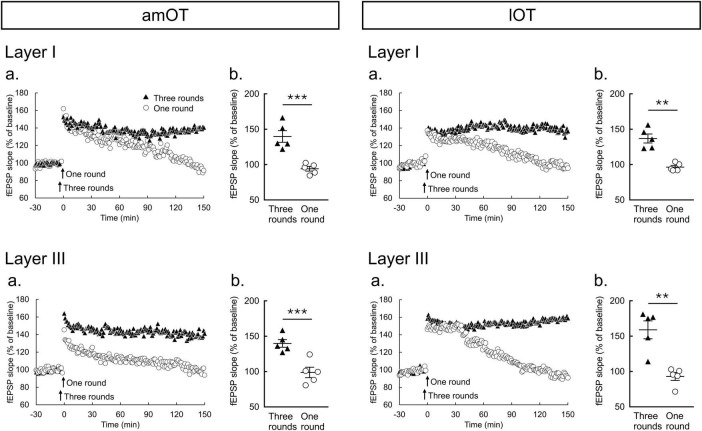
Two-hertz burst stimulation induces LTP in the amOT and lOT. **(a)** Time courses of mean fEPSP slopes (*n* = 5 brain slices from 5 mice in each condition of am/lOT layers with one or three round(s) of 2-Hz burst stimulation); **(b)** Comparisons of fEPSP slopes averaged at 140-150 min between one and three rounds of 2-Hz burst stimulation (unpaired *t*-test: **p < 0.01, ***p < 0.001). The bars in the columns indicate the mean ± SEM.

**FIGURE 4:**
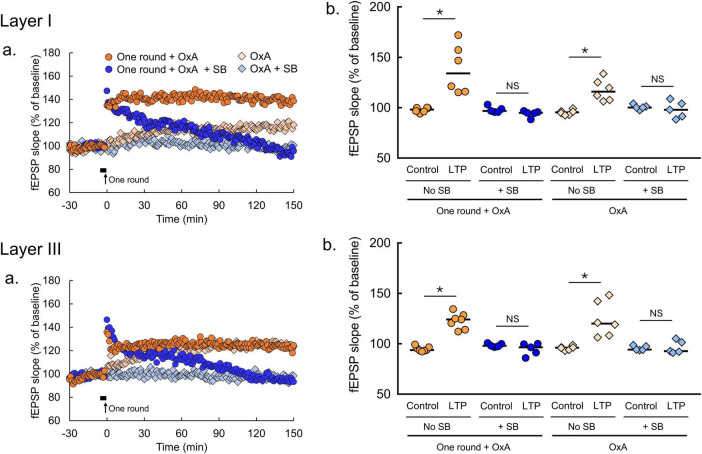
Orexin-A induces LTP in the amOT. **(a)** Time courses of mean fEPSP slopes (one round + OxA, *n* = 6 brain slices from 6 mice in layer I, *n* = 7 brain slices from 7 mice in layer III; one round + OxA + SB, *n* = 5 brain slices from 5 mice in layer I, *n* = 5 brain slices from 5 mice in layer III; OxA, *n* = 6 brain slices from 6 mice in layer I, *n* = 5 brain slices from 5 mice in layer III; OxA + SB, *n* = 5 brain slices from 5 mice in layer I, *n* = 5 brain slices from 5 mice in layer III). Filled horizontal bars, the drug application for 5 min; arrows, the onset of 2-Hz burst stimulation. **(b)** Comparisons of fEPSP slopes averaged at -30—20 min (control) with those at 140-150 min (LTP; Wilcoxon matched-pairs signed rank test: *p < 0.05, NS, not significant). OxA, bath application of orexin-A (100 nM); SB, bath application of SB334867 (3 μM); one round, one round of 2-Hz burst stimulation on the indicated layer. The bars in the columns indicate median.

**FIGURE 5:**
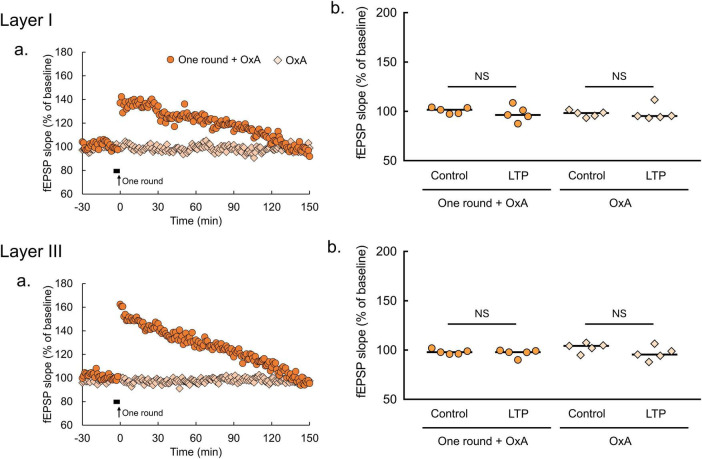
Orexin-A has no effect on fEPSP in the lOT. **(a)** Time courses of mean fEPSP slopes (*n* = 5 brain slices from 5 mice in each condition of lOT layers and with or without 2-Hz burst stimulation). Filled horizontal bars, drug application for 5 min; arrows, the onset of 2-Hz burst stimulation. **(b)** Comparisons of fEPSP slopes averaged at –30 and –20 min (control) with those at 140-150 min (LTP; Wilcoxon matched-pairs signed rank test: NS, not significant). OxA, bath application of orexin-A (100 nM); one round, one round of 2-Hz burst stimulation on the indicated layer. The bars in the columns indicate median.

To induce EPSPs at synapses in the OT, a stainless steel concentric bipolar stimulating electrode (Inter Medical, Nagoya, Japan) was placed on either layer I or III of the OT to stimulate input axons from other brain regions such as the olfactory bulb and various cortical and subcortical areas (Figure 1a). For field EPSP (fEPSP) recording, a glass microelectrode filled with normal ACSF (1-2 MΩ) was positioned on the same layer of the OT as the stimulating electrode. An electrical pulse (0.05-0.15 mA, duration of 50 μs) was applied every 30 s to elicit population spikes of the input axons to the OT (Figure 2, field-potential component 1), followed by fEPSP (Figure 2, field-potential component 2), in which the pulse intensity was adjusted so that the maximum initial slope (fEPSP slope) became 40-50% of the saturated fEPSP slope. The evoked field potentials were recorded, digitized at 10 kHz, and analyzed using the PowerLab/4sp system with Scope software (ADInstruments, Castle Hill, NSW, Australia). Using the system, two traces were averaged into a 1-min record and the fEPSP slope was measured to monitor synaptic efficacy throughout the recording. For LTP induction, 2-Hz burst stimulation was applied with the same intensity and pulse duration as the test stimulus. One round of 2-Hz burst stimulation consisted of 10 pulses at 100 Hz repeated at 2 Hz for 5 s, which was repeated three times at 175-s intervals for three rounds of 2-Hz burst stimulation. The burst frequency of 2 Hz was based on the results of our pilot study that tetanic or theta (5 Hz) burst stimulation frequently used in other brain regions failed to induce LTP in the OT, whereas reducing the bursting frequency from 5 to 2 Hz enabled LTP induction. The fEPSP slopes were normalized by baseline values, i.e., mean values of those for 10 min prior to 2-Hz burst stimulation or bath application of drug. LTP was evaluated at 140-150 min after the electrical stimulation and/or bath application of drug, similar to our previous studies using the olfactory bulb (Namba et al., 2016; Tong et al., 2017). The following numbers of animals were used in this study: fEPSP characterization, 1 mouse (Figure 2) and 2 mice (Supplementary Figure 1) in each layer of am/lOT; LTP induction by 2-Hz burst stimulation (Figure 3), 5 mice in each condition of am/lOT layers and one or three round(s) of 2-Hz burst stimulation; orexin action in the amOT (Figure 4), for one round of 2-Hz burst stimulation + orexin-A, 6 mice in layer I and 7 mice in layer III, for one round of 2-Hz burst stimulation + orexin-A + SB334867, 5 mice in each layer, for orexin-A, 6 mice in layer I and 5 mice in layer III, for orexin-A + SB334867, 5 mice in each layer, for ACSF, 3 mice in each layer (Supplementary Figure 2); orexin action in the lOT (Figure 5), for each condition of layers I/III and with or without 2-Hz burst stimulation, 5 mice, for one round of 2-Hz burst stimulation + SB334867, 3 mice (Supplementary Figure 3), for SB334867, 3 mice (Supplementary Figure 3).

### 2.4 Histochemistry

After the electrophysiological recording, the OT slices were stained with DAPI to identify the layer structures of the OT and the electrode locations (Figure 1b). The OT slices of 300 μm thick were fixed overnight in 4% paraformaldehyde at 4°C, rinsed 3 times with PBS for 15 min, stained with DAPI (1:500 diluted in PBS) for 15 min at room temperature. The stained preparations were observed under the upright fluorescent microscope with the monochrome digital camera (DM6000B, Leica, Wetzlar, Germany) and MetaMorph (Molecular Devices, CA, USA) to acquire the digitized images.

### 2.5 Chemicals

The reagents in this study including the α-amino-3-hydroxy-5 methyl-4-isoxazolepropionic acid (AMPA) and kainate receptor antagonist 6-cyano-7-nitroquinoxaline-2,3-dione (CNQX, 30 μM), the N-methyl-D-aspartate (NMDA) receptor antagonist DL-amino-5-phosphonopentanoate (AP-5, 50 μM), and the voltage-gated sodium channel blocker tetrodotoxin (TTX, 1 μM) were purchased from Sigma Aldrich (St. Louis, MO, USA). The orexin receptor agonist orexin-A (100 nM) and OxR1 antagonist SB334867 (3 μM) were purchased from Tocris Bioscience (Bristol, UK). All other reagents were purchased from FUJIFILM Wako Pure Chemical Corporation (Osaka, Japan). Orexin-A binds both OxR1 and OxR2 with similar affinity (Scammell and Winrow, 2011). The concentration of orexin-A used in this study was based on previous reports on the hippocampus (Selbach et al., 2004; Lu et al., 2016) and the ventral tegmental area (Borgland et al., 2006).

### 2.6 Statistical analysis

In LTP experiments, we used GraphPad Prism (GraphPad Software, San Diego, CA, USA) to estimate significant differences, which were indicated by *p* < 0.05. In Figure 3, unpaired *t*-test was applied for comparisons between one and three round(s) of 2-Hz burst stimulation at 140-150 min after the burst stimulation according to the results of Shapiro-Wilk test and *F*-test (Supplementary Table 1). In Figures 4, 5, Wilcoxon matched-pairs signed rank test was applied for comparisons between at −30 and −20 min and 140-150 min after the burst stimulation and/or bath application of drug as Shapiro-Wilk test rejected the null hypothesis of normality in some experimental groups (Supplementary Table 1). For sample size suitability in Figures 3–5, we performed sample size calculation using G*power software (Heinrich-Heine-Universität Düsseldorf, Düsseldorf, Germany, Supplementary Table 2).

## 3 Results

### 3.1 OT field potentials

First, the basic properties of field potentials in response to electrical stimulation were examined in layers I and III of the amOT and lOT. Layer I contains axons from the olfactory bulb and olfactory cortical areas including the piriform cortex, and layer III contains axons from olfactory cortical areas and presumably from other cortical and subcortical areas (Heimer, 1978; Luskin and Price, 1983; Wesson and Wilson, 2011; Sha et al., 2023). Representative evoked field potentials are shown in Figure 2 (*n* = 1 brain slice from 1 mouse in each layer of the am/lOT) and Supplementary Figure 1 (*n* = 2 brain slices from 2 mice in each layer of the am/lOT). In all recordings without drug application (control), weak negativity (field-potential component 1) was observed after the stimulus artifact. This weak negativity was followed by two slow deflections (field-potential components 2 and 3) in layer I and by one deflection (field-potential component 2) in layer III. AP-5 did not affect the response. CNQX abolished the slow deflections while maintaining the weak negativity. TTX eliminated the weak negativity and subsequent slow deflections.

These pharmacological experiments demonstrated that the weak negativity (component 1) was a population spike of the input fibers to the OT, and component 2 was a nonNMDA receptor-mediated fEPSP. Considering the OT neural circuits and onset latency (Owen and Halliwell, 2001; Carriero et al., 2009; Wieland et al., 2015), component 2 was regarded as a monosynaptic fEPSP induced *via* direct inputs from the olfactory bulb and other brain areas. Component 3 in layer I was considered a multisynaptic fEPSP evoked by the input from other olfactory cortical areas in response to layer I stimulation.

The wave form characteristics and pharmacological properties of field-potential components 1 and 2 were similar in layers I and III of the amOT and lOT. In the subsequent analyses of synaptic plasticity, we focused on component 2, the monosynaptic fEPSP elicited in response to electrical stimulation.

### 3.2 LTP induction by 2-Hz burst stimulation in the OT

To examine the plastic properties at synapses in the OT, the effects of 2-Hz burst stimulation on LTP induction were tested. In both layers I and III of the amOT and lOT, one round of 2-Hz burst stimulation induced short-term potentiation of the synaptic responses that decayed back to their baseline values, whereas three rounds of stimulation induced LTP that was maintained for 150 min (Figure 3a). The magnitudes of fEPSP slopes at 140-150 min were significantly larger for three rounds of stimulation than for one round of stimulation (Figure 3b). Although the mean magnitudes after one round of stimulation at 140-150 min were 93%-99% of the baseline values, those after three rounds of stimulation were 140% in layers I and III of the amOT (*p* = 0.0009 in layer I, *p* = 0.0021 in layer III, compared with one round of stimulation, unpaired *t*-test), respectively, and 137% and 159% in layers I and III of the lOT (*p* = 0.0004 in layer I, *p* = 0.0014 in layer III, compared with one round of stimulation, unpaired *t*-test), respectively. These observations indicated that LTP can be induced in the OT synapses, and the properties of synaptic plasticity in response to electrical stimulation in this study were similar between layers I and III in both the amOT and lOT.

### 3.3 Effects of orexin on LTP in the amOT and lOT

To determine the effects of orexin on synaptic plasticity in the OT, orexin-A was applied. In the amOT, bath application of orexin-A (100 nM) for 5 min induced LTP (Figure 4a). In both layers I and III, the magnitudes of fEPSP slopes at 140-150 min (LTP) were significantly larger than the baseline (control) values (OxA, *p* = 0.0313 in layer I, *p* = 0.0313 in layer III, Wilcoxon matched-pairs signed rank test, Figure 4b). Orexin-A-induced LTP in the amOT was abolished in the presence of SB334867 (OxA + SB, *p* = 0.8125 in layer I, *p* = 0.8125 in layer III, Wilcoxon matched-pairs signed rank test, Figure 4b). In addition, the enduring LTP was also induced in the amOT when subthreshold 2-Hz burst stimulation (one round) was paired with orexin-A application (one round + OxA, *p* = 0.0313 in layer I, *p* = 0.0156 in layer III, Wilcoxon matched-pairs signed rank test, Figure 4b). The effect of orexin-A was eliminated in the presence of SB334867 (one round + OxA + SB, *p* = 0.1250 in layer I, *p* = 0.3125 in layer III, Wilcoxon matched-pairs signed rank test, Figure 4b). Stable fEPSP recordings for 180 min under ACSF perfusion were confirmed by showing no differences in fEPSP slopes between −30 and −20 min and 140-150 min (Supplementary Figure 2).

By contrast, these effects of orexin-A were not apparent in the lOT (Figure 5a). Orexin-A application did not affect fEPSP slopes (OxA, *p* > 0.9999 in layer I, *p* = 0.6250 in layer III, Wilcoxon matched-pairs signed rank test, Figure 5b). Similarly, pairing of subthreshold 2-Hz burst stimulation (one round) and orexin-A application had no effect on fEPSP slopes (one round + OxA, *p* = 0.3125 in layer I, *p* = 0.1250 in layer III, Wilcoxon matched-pairs signed rank test, Figure 5b). SB334867 application also had no effect on fEPSP in the lOT (Supplementary Figure 3), arguing against the possibility that endogenous orexin in the lOT occluded the effect of exogenous orexin-A application. These results indicate that orexin-A promotes synaptic plasticity *via* OxR1 specifically in the amOT, but not in the lOT.

## 4 Discussion

### 4.1 NonNMDA receptor-mediated fEPSP and its activity-dependent long-lasting LTP at synapses in the mouse OT

Our recordings in the mouse OT indicated that the main component of field potentials induced by electrical stimulation of synaptic inputs in layers I and III was nonNMDA receptor-mediated fEPSP. This is consistent with the anatomical evidence that layer I includes glutamatergic axons from the olfactory bulb and olfactory cortical areas, whereas layer III includes glutamatergic axons from olfactory cortices and other cortical areas (Heimer, 1978; Luskin and Price, 1983; Wesson and Wilson, 2011; Sha et al., 2023). These properties of field potentials are consistent with those reported previously in the OT of rats (Owen and Halliwell, 2001), guinea pigs (Carriero et al., 2009), and mice (Wieland et al., 2015).

In the present analyses of synaptic plasticity, the characteristics of LTP induction by electrical axonal input stimulation were shared between layers I and III in the amOT and lOT. Three rounds of 2-Hz burst stimulation produced LTP that lasted for 150 min in the layers of the amOT and lOT. Previous studies found that LTP is induced by electrical burst stimulation at synapses in the piriform cortex, the largest area of the olfactory cortex (Kanter and Haberly, 1990; Poo and Isaacson, 2007; Morrison et al., 2013). Our results demonstrated that activity-dependent long-lasting LTP can also occur in the OT. Considering the general understanding that the mechanisms of long-lasting LTP in the brain include new protein synthesis to strengthen synaptic structures (Pfeiffer and Huber, 2006), the plastic property of the OT synapses can explain our recent observation that the synaptic ultrastructure of the OT develops when activation of the sensory or intracortical inputs to the OT was associated with reward or punishment (Sha et al., 2023).

The condition of electrical burst stimulation was adopted from our pilot study that tetanic or theta (5 Hz) burst stimulation, which is frequently used in other brain regions, failed to induce LTP in the OT, but reducing the bursting frequency to 2 Hz enabled LTP induction. The effectiveness of 2-Hz stimulation could reflect that the respiratory rhythm, closely related to olfaction, is critical for LTP induction at synapses in the OT. The rhythm of 2 Hz is within the lower range of the mouse breathing rate. Respiratory patterns are tightly related to animals’ behavior (Janke et al., 2022), and respiration-related oscillations are observed in the olfactory system, including the olfactory bulb, OT and piriform cortex and in many other brain areas including hippocampus and prefrontal cortex, which likely contribute to memory function (Carlson et al., 2014; Heck et al., 2019). Possible relationships among animals’ behavior, respiration, memory formation, and synaptic plasticity in the OT could be important for understanding the experience-dependent acquisition of motivated behaviors.

### 4.2 Orexin facilitates LTP in the amOT

Orexin accelerates LTP development in the amOT in an OxR1-dependent manner (Figure 4), but this was not replicated in the lOT (Figure 5). This domain-specific modulation by orexin reflects the higher expression of OxR1 in the amOT (Nogi et al., 2020), the crucial domain for odor-guided attraction (Murata et al., 2015). Glutamatergic transmission in the rodent OT is modified by acetylcholine (Hadley and Halliwell, 2010), serotonin (Owen and Halliwell, 2001), and dopamine (Wieland et al., 2015). In addition to these neurotransmitters, orexin can also modulate glutamatergic transmission in the mouse OT. Orexin ameliorates synaptic transmission efficiency in the CA1 and dentate gyrus of the hippocampus to induce LTP (Deadwyler et al., 2007; Zhao et al., 2014). Antagonizing OxR1 in the dentate gyrus impairs LTP induction (Akbari et al., 2011). Similarly, as observed in the hippocampus, our study revealed the effects of orexin on LTP induction *via* OxR1 signaling at glutamatergic transmission in the amOT. This action of orexin might contribute to the structural development of synaptic inputs to the amOT and activation of the amOT by odor-food reward association learning (Murata et al., 2015; Sha et al., 2023).

Given that glutamatergic transmission in the OT includes peripheral inputs from the olfactory bulb and central inputs from the piriform cortex, one possibility is that orexin activates OxR1 expressed on the postsynaptic side of glutamatergic transmission in the amOT to modulate the synaptic efficacy. Previous investigations in rats reported that orexin acts on the postsynaptic side of the hippocampus CA1 neurons to modify LTP (Aou et al., 2003) and that of the prefrontal cortex to excite pyramidal neurons (Xia et al., 2005). The other possibility is that orexin binds to OxR1 expressed at the presynaptic side of glutamatergic transmission in the amOT to modulate glutamate release. A previous study in mice demonstrated that orexin stimulates neurons in part by promoting glutamate release from the presynaptic axon terminals of interneurons to neurons in the lateral hypothalamus (Li et al., 2002). Further experiments such as *in situ* hybridization of OxR1 and patch-clamp recording of OT neurons would elucidate the mechanisms of action of orexin at glutamatergic synapses in the amOT.

The present study identified the contribution of glutamatergic inputs and neuromodulatory signals in the synaptic plasticity of the OT. Combining these two factors is considered crucial for promoting OT domain-specific circuit plasticity and acquiring odor valences in a learning-dependent manner. Further understanding of this combinatory role could facilitate the neural circuit mechanisms of adaptive learning of odor-guided motivated behaviors.

## Data Availability

The original contributions presented in this study are included in this article/Supplementary material, further inquiries can be directed to the corresponding authors.
