# Supplementary material for: Synaptic plasticity and roles of orexin in distinct domains of the olfactory tubercle
Source: Front Neural Circuits. 2024 Nov 7;18:1473403. doi: 10.3389/fncir.2024.1473403 (PMC11578722; doi:10.3389/fncir.2024.1473403)
Supplement: Supplementary file 1 [file Data_Sheet_1.pdf]

Supplementary material

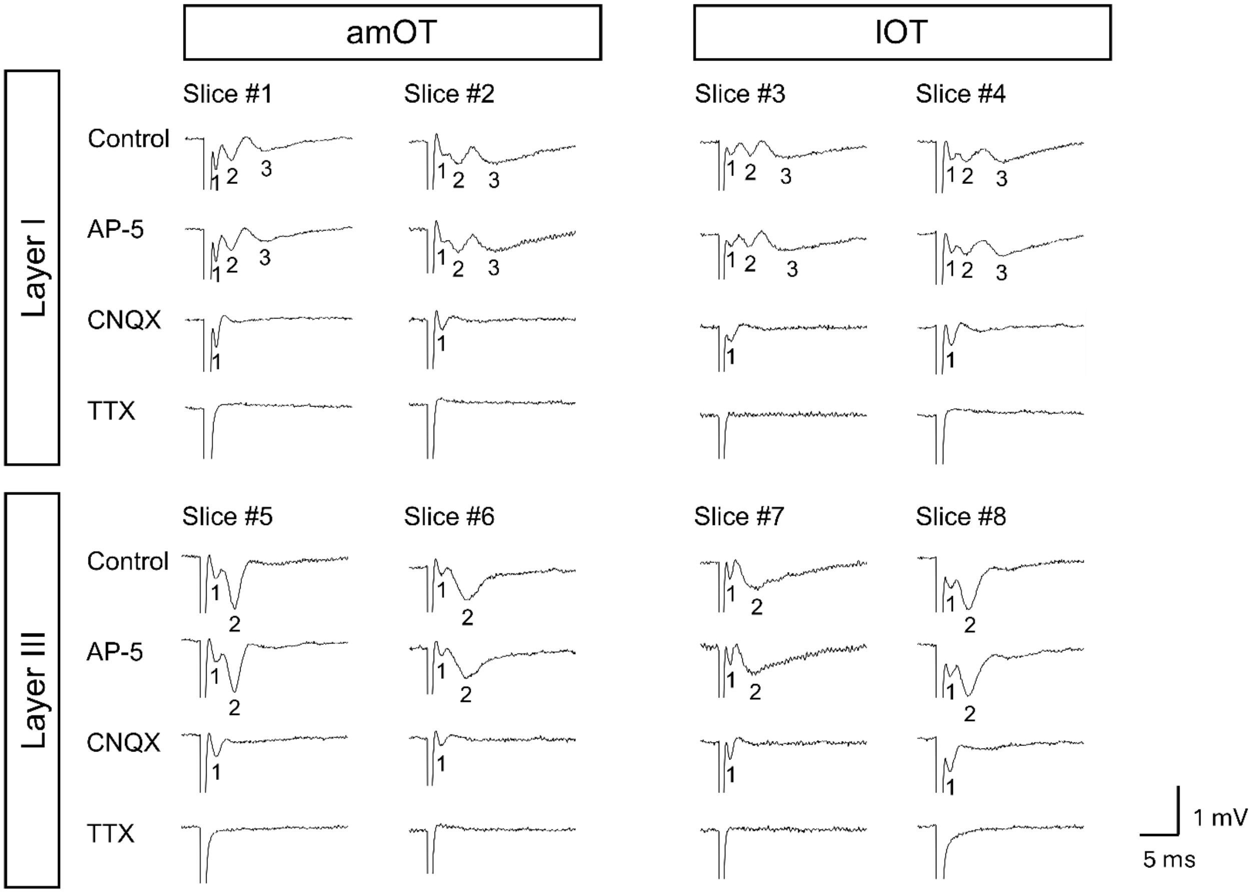

Figure S1. Examples of evoked field potentials in the OT recorded in brain slices different from those in Figure 2. Each brain slice was obtained from an individual mouse. As observed in Figure 2, the recording traces in the control present the weak negativity (field-potential component 1) followed by one (layer III) or two (layer I) slow deflections (field-potential components 2 and 3). Bath application of drug: NMDA receptor antagonist AP-5 (50 µM), AMPA and kainate receptor antagonist CNQX (30 µM), or voltage-gated sodium channel blocker TTX (1 µM).

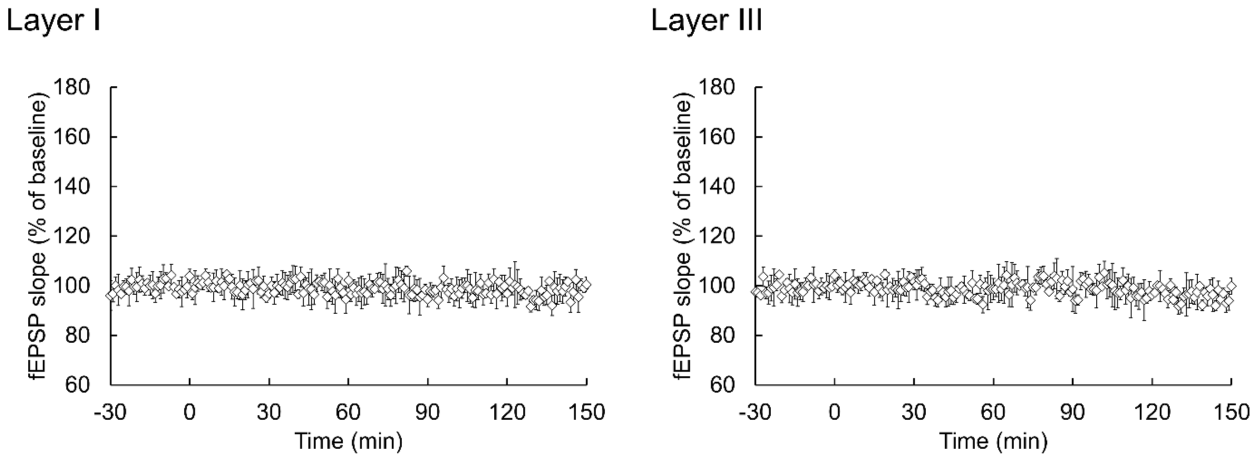

Figure S2. Baseline recordings of fEPSP for 180 min under ACSF perfusion. Time courses of fEPSP slopes (mean ± SD) in the amOT: n = 3 brain slices from 3 mice in layer I (left), n = 3 brain slices from 3 mice in layer III (right).

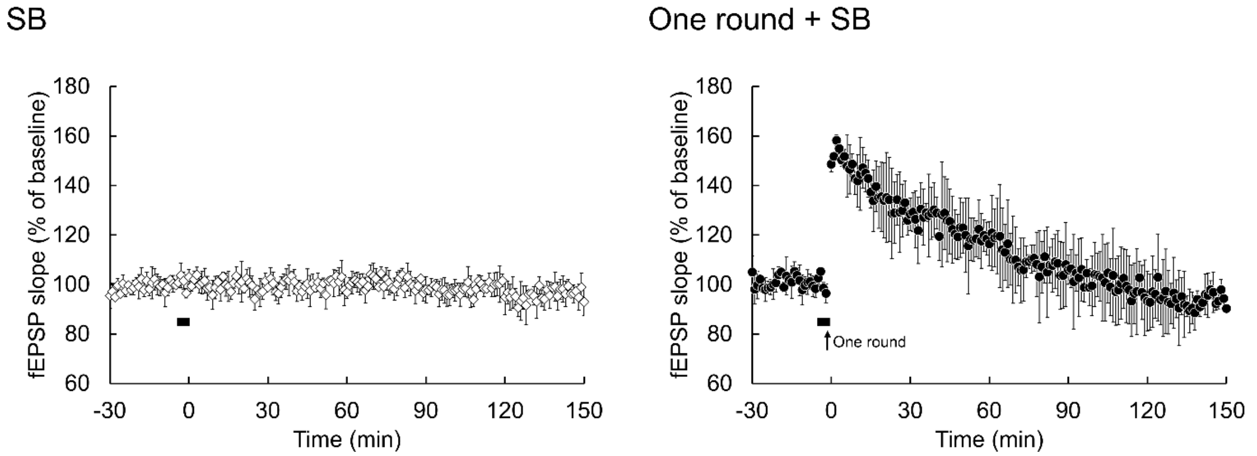

Figure S3. SB334867 application has no effect on fEPSP in the lOT. Time courses of fEPSP slopes (mean ± SD) in layer III: SB (n = 3 brain slices from 3 mice), one round + SB (n = 3 brain slices from 3 mice). Filled horizontal bars, drug application for 5 min; arrow, the onset of 2-Hz burst stimulation; SB, bath application of SB334867 (3 µM); one round, one round of 2-Hz burst stimulation.

Table S1. Normality and variance test by GraphPad Prizm using the data obtained in this study

Data in Figure 3b

Layer			n	Shapiro-Wilk test		*F*-test		
				p value		*F* value	*p* value	
amOT	I	3 rounds	5	0.3625	NS	6.312	0.1020	NS
		1 round	5	0.7581	NS			
	III	3 rounds	5	0.6987	NS	1.819	0.5766	NS
		1 round	5	0.6598	NS			
lOT	I	3 rounds	5	0.5152	NS	6.606	0.0946	NS
		1 round	5	0.1970	NS			
	III	3 rounds	5	0.1360	NS	5.075	0.1447	NS
		1 round	5	0.1032	NS			

NS, not significant.

Data in Figures 4b and 5b

	Layer			n	Shapiro-Wilk test *p* value	
amOT	I	1 round + OxA	Control	6	0.2163	NS
			LTP		0.2587	NS
		1 round + OxA + SB	Control	5	0.1393	NS
			LTP		0.0669	NS
		OxA	Control	6	0.7296	NS
			LTP		0.5965	NS
		OxA + SB	Control	5	0.7660	NS
			LTP		0.7819	NS
	III	1 round + OxA	Control	7	0.3644	NS
			LTP		0.8621	NS
		1 round + OxA + SB	Control	5	0.6003	NS
			LTP		0.3952	NS
		OxA	Control	6	0.7658	NS
			LTP		0.2975	NS
		OxA + SB	Control	5	0.1310	NS
			LTP		0.1345	NS
lOT	I	1 round + OxA	Control	5	0.2953	NS
			LTP		0.9652	NS
		OxA	Control	5	0.9078	NS
			LTP		0.0034	**
	III	1 round + OxA	Control	5	0.3772	NS
			LTP		0.9254	NS
		OxA	Control	5	0.4338	NS
			LTP		0.0226	*

Significant at *$p < 0.05$, **$p < 0.01$. NS, not significant.

Table S2. Sample size calculation computed by G*power using the data obtained in this study

Data in Figure 3b

	Layer			n	Mean	SD	Allocation (N2/N1)	Calculated sample size
amOT	I	3 rounds	***	5 (N1)	139.8	18.5	1	8
		1 round		5 (N2)	94.1	7.3		8
	III	3 rounds	**	5 (N1)	139.8	12.3	1	8
		1 round		5 (N2)	98.8	16.5		8
lOT	I	3 rounds	***	5 (N1)	136.9	14.3	1	8
		1 round		5 (N2)	96.4	5.5		8
	III	3 rounds	**	5 (N1)	158.9	28.3	1	7
		1 round		5 (N2)	92.9	12.6		7

Unpaired *t*-test performed in Figure 3b: significant at $^{**}p < 0.01$, $^{***}p < 0.001$. Sample size calculation: α error probability = 0.05, power (1 - β probability) = 0.95.

Data in Figures 4b and 5b

	Layer			n	Mean of difference	SD of difference	Calculated sample size
amOT	I	1 round + OxA	*	6	40.3	25.5	8
		1 round + OxA + SB	NS	5	-3.9	6.1	36
		OxA	*	6	22.1	10.6	6
		OxA + SB	NS	5	-2.5	4.7	51
	III	1 round + OxA	*	7	27.9	7.7	4
		1 round + OxA + SB	NS	5	-3.9	6.3	38
		OxA	*	6	28.4	16.3	7
		OxA + SB	NS	5	0.91	6.4	697
lOT	I	1 round + OxA	NS	5	-3.2	6.7	62
		OxA	NS	5	0.5	6.1	2026
	III	1 round + OxA	NS	5	-6.1	5.5	14
		OxA	NS	5	-1.3	3.2	85

Wilcoxon matched-pairs signed rank test performed in Figures 4b and 5b: *significant at $p < 0.05$; NS, not significant. Sample size calculation: α error probability = 0.05, power (1 - β probability) = 0.95.
